# Successful Use of Dupilumab as a Salvage Therapy for Recalcitrant Allergic Fungal Rhinosinusitis: A Case Report

**DOI:** 10.7759/cureus.23104

**Published:** 2022-03-12

**Authors:** Revan A Mujahed, Osama A Marglani, Lama S Maksood, Talah A Felemban

**Affiliations:** 1 College of Medicine, Umm Al-Qura University, Makkah, SAU; 2 Otolaryngology-Head and Neck Surgery, Umm Al-Qura University, Makkah, SAU; 3 Otolaryngology-Head and Neck Surgery, King Faisal Specialist Hospital and Research Centre, Jeddah, SAU

**Keywords:** afrs, monoclonal antibody, refractory, otolaryngology, chronic rhinosinusitis, allergic fungal rhinosinusitis, duplimab

## Abstract

Allergic fungal rhinosinusitis (AFRS) is a subtype of chronic rhinosinusitis with nasal polyps (CRSwNP) which is distinguished by the presence of eosinophilic mucin, type 1 hypersensitivity reaction resulting from fungi residing within the sinus, and characteristic imaging findings of the paranasal sinuses. Surgical intervention, sinonasal irrigations, and topical and systemic medications are commonly used to reduce the fungal load and antigenic stimulation. Despite the advancement of medical and surgical management of AFRS, a high recurrence rate is still a significant concern. The proper treatment for refractory AFRS remains controversial. Herein, we discuss the use of dupilumab for controlling refractory AFRS. We report a case of a 33-year-old female patient known to have had AFRS for 16 years. Due to the recurring nature of her illness, 16 functional endoscopic sinus surgeries (FESS) have been done to control her symptoms. The last operation was done in our institution; evidence for cure was insufficient with the persistence of symptoms. After a consensus decision with the multidisciplinary management team, she was an appropriate candidate for therapy with dupilumab. After six months of using the medication, magnificent improvement and control of symptoms were noted, and post-treatment CT scans illustrated excellent progression from previous scans. AFRS could be an extremely debilitating disease with significant impairment of quality of life even when standard therapy and extensive surgical interventions are implemented. Dupilumab can be an excellent option as a salvage therapy for recalcitrant AFRS with significant improvement in patients' quality of life and resolution of symptoms.

## Introduction

Chronic rhinosinusitis (CRS) is a heterogeneous group of sinonasal disorders that can be classified into two broad categories: chronic rhinosinusitis without nasal polyps (CRSsNP) and chronic rhinosinusitis with nasal polyps (CRSwNP) [1,2]. Allergic fungal rhinosinusitis (AFRS) is a subtype of chronic rhinosinusitis with nasal polyps which is characterized by the presence of eosinophilic mucin (i.e., allergic mucin), type 1 hypersensitivity reaction resulting from fungi residing within the sinus, and characteristic imaging findings of the paranasal sinuses [1,2]. AFRS is not an uncommon disease as it accounts for 6-9% of all rhinosinusitis cases [3]. AFRS commonly affects patients younger than those affected by other forms of CRS and with more female predominance. Some factors have been described in the literature to be associated with AFRS occurrences, such as geographical region, climate, and certain host-related factors, specifically lower socioeconomic demographic and warm climate in addition to African American ethnicity [1,2]. It is also worth mentioning that this patient group tends to have other atopic conditions such as allergic rhinitis and childhood asthma [2]. Despite the advancement of medical and surgical management of AFRS, a high recurrence rate is still a significant concern, with a reported rate of around 50-60% in multiple previous studies [4-6]. AFRS is an allergic disease in which the non-invasive fungal colonization induces hypersensitivity reactions [7]. Therefore, there is sound reasoning to use biological agents that target eosinophilic inflammation or type two inflammation-driven disease [1]. This case report aimed to represent the effect of dupilumab, an interleukin-4 (IL-4) receptor antagonist, on the number of relapse episodes in a case of refractory AFRS and the associated parameters. The patient gave informed consent regarding the publication of this case report.

## Case presentation

We report a case of a 33-year-old female referred to our institution for refractory allergic fungal rhinosinusitis for 16 years. Before presenting to our care, she had undergone 15 functional endoscopic sinus surgeries (FESS) and reported using corticosteroid for a prolonged period since diagnosis. The patient complained of left facial swelling, retro-orbital pain, headache, sneezing, nasal obstruction, greenish purulent nasal discharge, hyposmia, and impaired vision in the left eye. Her allergic history is broad and encompasses multiple allergens, including banana, strawberry, shrimp, egg, kiwi, acetylsalicylic acid, diclofenac, in addition to significant asthma-like symptoms. 

**Figure 1:**
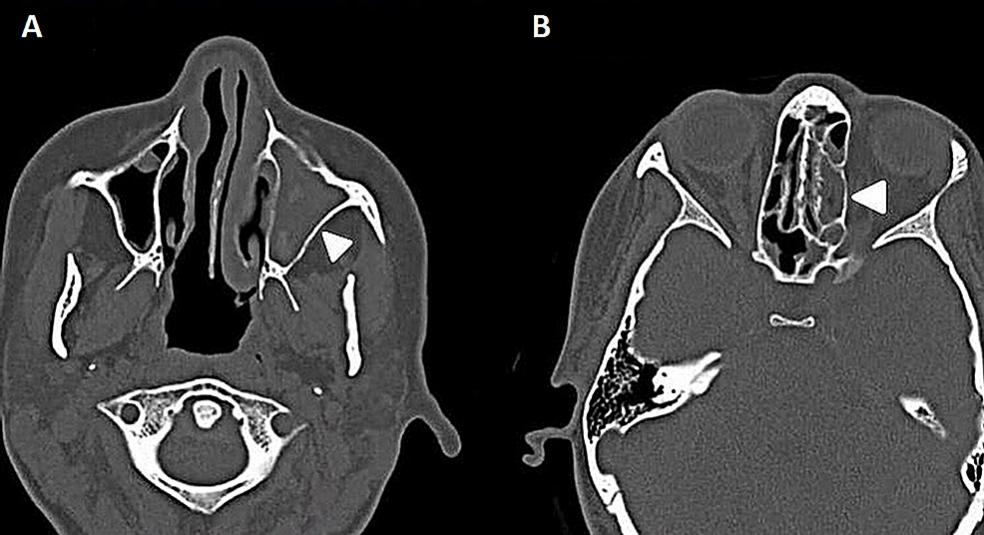
Pre-treatment CT scans showing persistent partial opacification of the paranasal sinuses with high internal densities. (A) Almost complete opacification of the left maxillary sinus prior to medication use (arrowhead); (B) mucosal thickening of ethmoid sinuses more marked on the left side prior to medication use (arrowhead).

Upon examination, the endoscopy revealed a congested mucosa with purulent discharge and thick mucus production with no polyps. Computed tomography (CT) scan showed complete opacification in the left maxillary sinus and hyperdense material suggestive of fungal sinusitis. Moreover, bilateral ethmoid sinus opacification was detected, and the right maxillary was also involved to a lesser degree. The patient underwent the 16th functional endoscopic sinus surgery two years ago in our hospital in January 2019. The histopathology sample showed inflammatory nasal polyp unfavorable for a fungal organism, dysplasia, or malignancy. The patient's symptoms progressively returned over two years and five months post-operative during follow-up. Firstly, nasal obstruction occurred after one month, headache and rhinorrhea after seven months, hyposmia after a year, and scope examination indicated significant left maxillary polyps limited to the ostia, while the left frontal, sphenoid, ethmoid sinuses, and right sinuses were all patent. Her symptoms continued when she presented after one year and seven months, exhibiting facial pressure and eye symptoms. On examination with a scope, the left side revealed clear mucin. Pre-treatment CT scans were performed in November 2020 and showed persistent partial opacification of the paranasal sinuses with high internal densities, which could be related to inspissated secretions with or without fungal colonization (Figures 1A, 1B). The disease progressed mainly within the left maxillary sinus and the left ethmoid sinus was also involved. 

**Figure 2:**
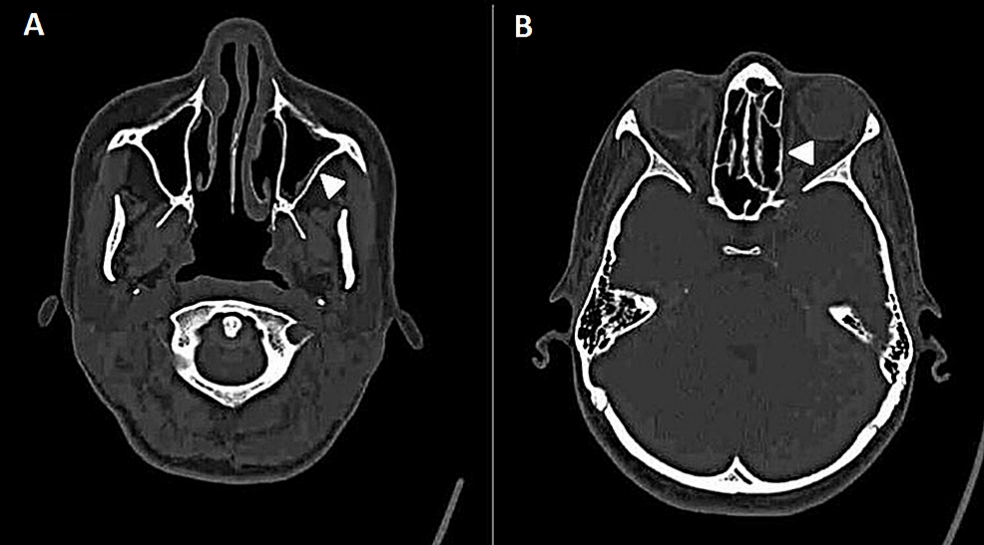
Post-treatment CT scans of the patient. (A) Post-medication total improvement of the opacified left maxillary sinus (arrowhead); (B) improved ethmoid sinuses post-medication use (arrowhead).

Since the onset of symptoms, the patient has been treated with oral steroids, normal saline nasal wash, mometasone nasal spray, and cetirizine with no improvement following a three-year follow-up period in our hospital, in which operative evidence for cure was insufficient with the persistence of symptoms. A consensus decision with the multidisciplinary management team, which included otolaryngologists and immunologists, she was an appropriate candidate for therapy with dupilumab 300 mg subcutaneous injection every two weeks. The patient showed magnificent improvement and control of symptoms after being on dupilumab for six months. Post-treatment CT scans were performed in August 2021 have illustrated excellent progression (Figures 2A, 2B). Sinonasal outcome test (SNOT-22) has declined from 93 prior to treatment to 21 post six months of treatment administration. The patient is satisfied with the results of the planned care with marked improvement in her quality of life. 

## Discussion

AFRS patients suffer from impaired quality of life compared to the general population, and their quality of life is much lower in patients with extensive and recurrent disease [8]. In the literature, the clinical presentation of AFRS patients varies from characteristic thick greenish-brown nasal discharge with a peanut butter-like consistency, nasal obstruction, anosmia, and nasal polyps. Without timely intervention, these patients present with a more complicated clinical picture which include visual disturbances, proptosis, facial deformity, and intracranial sequelae [1,7]. 

Surgical debridement to remove the antigenic stimulation for the disease constitutes the mainstay of AFRS treatment, followed by oral/topical corticosteroids to decrease recurrence post-surgery [2]. Other adjunctive pharmacological therapies such as topical or oral antifungals have been used with an insufficient evidence base for its effectiveness [2], and a Cochrane review of topical and systemic antifungal treatments for CRS patients did not indicate any clinical benefit [9].

IgE-mediated hypersensitivity reaction forms a part of the pathophysiology of AFRS development, but it is not the only component of the pathophysiological process as it exhibits multiple cellular immunological interactions [7]. It has been reported in the literature that dupilumab was successfully used for type 2 inflammation-driven diseases such as asthma, atopic dermatitis, and eosinophilic esophagitis [10-12]. These patients with AFRS tend to have a higher level of IgE antifungal antibodies in addition to eosinophil-rich mucus; both are type 2 responses that have been traditionally described to be caused by an exacerbated Th2 adaptive immune response [1]. In addition, IL-4 and IL-13 are inflammatory cytokines that play an essential role in type 2 inflammation responsible for the pathophysiology of AFRS [13]. 

In June 2019, the FDA approved dupilumab use for inadequately controlled CFSwNP, but there was no specification for its use in AFRS [14]. Dupilumab is an IL-4 alpha receptor blocker, so its net effect is blocking IL-4 and IL-13 [13]. Moreover, it suppresses the production of type 2 inflammatory markers in peripheral blood and nasal polyp tissue such as eosinophilic cationic protein (ECP), eotaxins, IgE, and IL-4 alpha-receptor inhibitor. Furthermore, dupilumab may also work by other mechanisms, including suppression of B-cell and IgE production and inhibiting inflammatory cellular trafficking in inflamed tissue through the endothelium [15,16].

In 2019, the European Forum for Research and Education in Allergy and Airway Diseases (EUFOREA) recommended criteria regarding the use of monoclonal antibodies for CRSwNP patients. The criteria consist of bilateral nasal polyps, history of sinus surgery in addition to three of the following five criteria: (1) evidence of type 2 inflammation, (2) impaired quality of life, (3) two or more corticosteroids courses used during past two years for disease control, (4) asthma diagnosis, and (5) significant loss of smell [17]. Therefore, dupilumab use for allergic fungal rhino-sinusitis - a type 2 inflammation disease - warrants a great response to the medication and is expected to be effective for resistant allergic fungal rhinosinusitis. The medication was successfully used in two previously reported resistant AFRS with significant efficacy and response [13,15]. 

Finally, after one year, the response to biological treatment may be evaluated using the EUFOREA defined criteria, which include the following: (1) reduction in the size of nasal polyps, (2) reduction in systemic corticosteroids use, (3) improvement in quality of life, (4) improvement in the sense of smell, and (5) decreased comorbidities impact. A good response is suggested when three to four criteria are met [17]. Treatment was well-tolerated during a six-month period, and our patient showed a significant response to the medication and substantial improvement in quality of life.

## Conclusions

Dupilumab can be an excellent medication for controlling refractory AFRS requiring recurrent surgical interventions. However, given the limited number of cases that have been successfully treated with the medication, it is not clear whether certain factors would affect the response to the medication. Therefore, further studies are needed to assess the factors that affect medication response on different patients and to describe such patients' clinical features and characteristics.
